# Effect of Intraperitoneal Chemotherapy with Regorafenib on IL-6 and TNF-α Levels and Peritoneal Cytology: Experimental Study in Rats with Colorectal Peritoneal Carcinomatosis

**DOI:** 10.3390/jcm12237267

**Published:** 2023-11-23

**Authors:** Stefanos Bitsianis, Ioannis Mantzoros, Elissavet Anestiadou, Panagiotis Christidis, Christos Chatzakis, Konstantinos Zapsalis, Savvas Symeonidis, Georgios Ntampakis, Kalliopi Domvri, Anastasia Tsakona, Chryssa Bekiari, Orestis Ioannidis, Stamatios Aggelopoulos

**Affiliations:** 1 4th Department of General Surgery, Aristotle University of Thessaloniki, General Hospital of Thessaloniki “G. Papanikolaou”, 54124 Thessaloniki, Greece; sbitsiani@gmail.com (S.B.); imanvol@gmail.com (I.M.); elissavetxatz@gmail.com (E.A.); panagiotischristidis13@gmail.com (P.C.); cchatzakis@gmail.com (C.C.); konstzapsalis@yahoo.gr (K.Z.); simeonidissavvas@yahoo.com (S.S.); gntampak@auth.gr (G.N.); saggelopoulos@auth.gr (S.A.); 2 Laboratory of Histology-Embryology, Medical School, Aristotle University of Thessaloniki, 54124 Thessaloniki, Greece; kellybio4@hotmail.com; 3 Department of Pathology, General Hospital of Thessaloniki “G. Papanikolaou”, 54124 Thessaloniki, Greece; 4 Pathology Department, Faculty of Medicine, Aristotle University of Thessaloniki, 54124 Thessaloniki, Greece; anastasiatsakona28@yahoo.com; 5 Experimental and Research Center, Papageorgiou General Hospital of Thessaloniki, 56403 Thessaloniki, Greece; chmpekia@vet.auth.gr; 6 Laboratory of Anatomy and Histology, Veterinary School, Aristotle University of Thessaloniki, 54124 Thessaloniki, Greece

**Keywords:** regorafenib, peritoneal carcinomatosis, intraperitoneal chemotherapy, cytoreductive surgery, colorectal cancer, intraperitoneal injection

## Abstract

Cytoreductive surgery (CRS), combined with hyperthermic intraperitoneal chemotherapy, has significantly improved survival outcomes in patients with peritoneal carcinomatosis from colorectal cancer (CRC). Regorafenib is an oral agent administered in patients with refractory metastatic CRC. Our aim was to investigate the outcomes of intraperitoneal administration of regorafenib for intraperitoneal chemotherapy (IPEC) or/and CRS in a rat model of colorectal peritoneal metastases regarding immunology and peritoneal cytology. A total of 24 rats were included. Twenty-eight days after carcinogenesis induction, rats were randomized into following groups: group A: control group; group B: CRS only; group C: IPEC only; and group D: CRS + IPEC. On day 56 after carcinogenesis, euthanasia and laparotomy were performed. Serum levels of interleukin-6 (IL-6) and tumor necrosis factor α (TNF-α) as well as peritoneal cytology were investigated. Groups B and D had statistically significant lower mean levels of IL-6 and TNF-α compared to groups A and C, but there was no significant difference between them. Both B and D groups presented a statistically significant difference regarding the rate of negative peritoneal cytology, when compared to the control group, but not to group C. In conclusion, regorafenib-based IPEC, combined with CRS, may constitute a promising tool against peritoneal carcinomatosis by altering the tumor microenvironment.

## 1. Introduction

Colorectal cancer (CRC) is the second major cause of death due to malignancy in the United States. Mortality associated with CRC is almost always the result of metastatic spread to the liver, the peritoneal cavity, or the lungs [1]. Peritoneal metastasis (PM) is synchronous with the primary tumor in 5% of patients during initial diagnosis and 25% of patients with stage IV disease who present at onset with peritoneal involvement [2]. However, the true incidence of PM is ambiguous, since peritoneal metastatic disease is under-diagnosed due to difficult detection with routine imaging protocols [1]. The presence of other organ involvement with PM is associated with shorter overall survival (OS) by 30–40% when compared to other sites of metastasis [3]. Median OS is only 5 months in untreated cases, while systemic chemotherapy slightly improves the median OS to 15 months [4].

Initially proposed by Sugarbaker, cytoreductive surgery (CRS) and hyperthermic intraperitoneal chemotherapy (HIPEC) can increase long-term survival in cautiously selected patients with peritoneal carcinomatosis from CRC; thus, the median OS range from 22 up to 63 months in specialized centers [5]. CRS for PM from CRC is aimed at removing all macroscopic nodules leaving no residual disease, since intraperitoneal chemotherapy (IPEC) is not effective in eradicating tumor implants larger than 2.5 mm [6]. In addition, IPEC includes the regional administration of chemotherapy of cytotoxic drugs in a concentrated dose, providing a higher cytotoxic effect to peritoneal tumor cells and low systemic cytotoxicity via localized delivery due to the plasma–peritoneal barrier [7]. What is more, HIPEC is based on the fact that antineoplastic agents above 41 °C present selective cytotoxicity to tumor cells, and increased absorption and penetration of the tumor tissue [8]. However, PRODIGE 7, a phase III clinical trial, suggested that in patients with PM from CRC, HIPEC was associated with a higher rate of complications, without improving the OS, when compared to cytoreductive surgery alone. In this way, more preclinical and clinical trials are necessary for the establishment of cytotoxic agents with a wider profile of safety and efficacy [9]. 

Among the cytotoxic agents that have recently emerged, regorafenib is an oral small-molecule multiple kinase inhibitor, blocking the activity of angiogenic, stromal, and oncogenic protein kinases, which are administered worldwide in heavily pre-treated patients with metastatic CRC [10]. The mechanism of cytotoxicity, through broad kinase, targets several crucial parts of CRC development, such as angiogenesis (by inhibiting VEGFR1, −2, −3, TIE2, PDGFR, and FGFR1 and −2), proliferation (by inhibiting c-KIT, RAF1, BRAF, and RET), metastasis (by inhibiting VEGFR2 and −3, and PDGFR), and immunosuppression (by inhibiting CSF1R) [11]. However, no preclinical or clinical studies have investigated the role of the intraperitoneal administration of regorafenib as a potential therapeutic agent in IPEC. 

Numerous inflammatory factors are expressed in the tumor microenvironment. Among them, interleukin-6 (IL-6) is an inflammatory marker with a pleiotropic effect [12]. IL-6 holds a key role in the immunization of CRC and is related to adverse prognosis [13]. Tumor necrosis factor α (TNF-α), originally known as cachectin, promotes the malignancy potential and adhesion of tumor cells to vascular endothelial or lymphatic endothelium, thus increasing lymphatic or blood-transfer metastasis [13]. Pro-inflammatory cytokines have a crucial role in CRC cachexia by inducing a Systemic Inflammatory Response (SIR), which leads to poor prognosis and an increased morbidity rate [12]. Moreover, IL-6 and TNF-a seem to play an important role in the PM cascade as they both enhance the expression of adhesion molecules, crucial for the attachment of intraperitoneal cancer cells to the distant peritoneum, and promote the angiogenesis necessary for the final step of PM, tumor proliferation [14].

Nowadays, peritoneal cytology is the standard test to confirm the diagnosis of micrometastatic peritoneal spread. While in gastric and ovarian cancer the role of peritoneal cytology is established, its importance in CRC remains unclear [15]. Multiple studies propose a relationship between cytology positivity, poor prognosis, and peritoneal recurrence [16]. However, there is a scarcity of data regarding the role of peritoneal cytology as a prognostic marker after CRS + HIPEC.

Based on the multi-targeted therapy of regorafenib, the aim of the present experimental study is to investigate the effects of IPEC with regorafenib, with or without CRS, in a metastatic CRC rat model regarding the serum cytokine profile, as well as to study ascites cytology among groups.

## 2. Materials and Methods

### 2.1. Protocol

This double-blind, randomized, prospective experimental study received approval by the ethical committee of the Veterinary Medicine Department of Central Macedonia in Greece (577170/2467, 3/11/2020). All necessary approved protocols for laboratory animal care were applied. The experiment was performed and results were published in accordance with the ARRIVE (Animal Research: Reporting of In Vivo Experiments) guidelines 2.0 [17].

### 2.2. Experimental Animals

Wistar rats were chosen because of their anatomical and biological similarities to humans regarding peritoneal metastasis from CRC, and because of the numerous previously published experimental models of IPEC in rats [18]. An experimental animal protocol was designed to minimize pain and discomfort in animals. A total of 24 Wistar rats, 10–14 weeks old, with weights of approximately 200–300 g, were individually housed in cages, under a controlled temperature of 18–22 °C and humidity of 55–60%, with controlled 12 h intervals of light and darkness. Rats had free access to water and food. No antibiotics were administered, and male rats were chosen to avoid the effects of hormone-related factors in the experiment.

### 2.3. Experimental Procedures

The HT-29 cell line is a human colorectal adenocarcinoma cell line with epithelial morphology. The experimental process began with in vitro cultivation of the HT-29 (ATCC^®^ HTB-38™) cell line, following the procedure described thoroughly in our previous study [19]. After this procedure, shots of 2 mL solutions with a concentration of 10^6^/mL cancer cells were prepared for administration.

At T0 (Day 0), rats underwent general anesthesia, with the administration of ketamine (50 mg/kg) and xylazine (5 mg/kg), and cancer cells were injected at the right lower quadrant of the abdomen and the mesentery of the cecum. Rats were kept in individual cages for a total of 28 days under the aforementioned circumstances and were administered analgesics during the first 3 postoperative days. At T1 (Day 28), the rats were randomly and equally allocated into 4 groups of 6 rats, with the use of an electronic randomization software. The groups of the study were formed as follows:

- Group A (control group): midline laparotomy was performed and isothermic normal saline was administered, without any other intervention.
- Group B (CRS group): midline laparotomy, right hemicolectomy, and cytoreductive surgery were performed.
- Group C (IPEC group): midline laparotomy was performed and intraperitoneal chemotherapy with regorafenib (BAY 73-4506, Selleck Chemicals, Houston, TX, USA) was administered.
- Group D (CRS + IPEC group): midline laparotomy, right hemicolectomy, and CRS were performed and IPEC with regorafenib was administered.

Rats were under general anesthesia during the experimental process. In groups B and D, CRS included peritonectomy, omentectomy, hepatic parenchyma metastasectomy, and removal of the mesentery foci when feasible, aiming at the radical resection of all macroscopic metastatic nodules. Cauterization with the use of an electrocoagulation device was performed in irresectable tumor deposits in sensitive regions such as the liver hilum, spleen hilum, diaphragm, and mesenterium in order to avoid additional stress. Apart from CRS, groups B and D were also treated with right hemicolectomy with end-to-side anastomosis. After CRS completion, residual disease was graded according to a classification system used in clinical practice (R0: no residual tumor, minimal distance between tumor and resection margin ≥ 1 mm; R1: no residual tumor, minimal distance between tumor and resection margin ≤ 1 mm; R2a: macroscopic residual tumor <2.5 mm; R2b: macroscopic residual tumor >2.5 mm) [18].

During the initial surgical procedure, 250 mL of isothermic normal saline 0.9% for group A and a solution containing regorafenib (10 mg/kg) dissolved in 250 mL of isothermic normal saline for groups C and D were prepared. In total, 10 mL of the prepared solution was infused every 2 min and then suctioned. After the complete administration of the solution, the peritoneal cavity was irrigated with 0.9% normal saline for 5 min. Regarding the cytotoxic agent dose, regorafenib was administered in a single dose at a concentration of 10 mg/kg for each rat, leading to exposures within the accepted range for a human dose of 160 mg [19]. In group D, combining CRS with IPEC, colonic anastomosis was performed after cytotoxic agent administration. 

**Figure 1:**
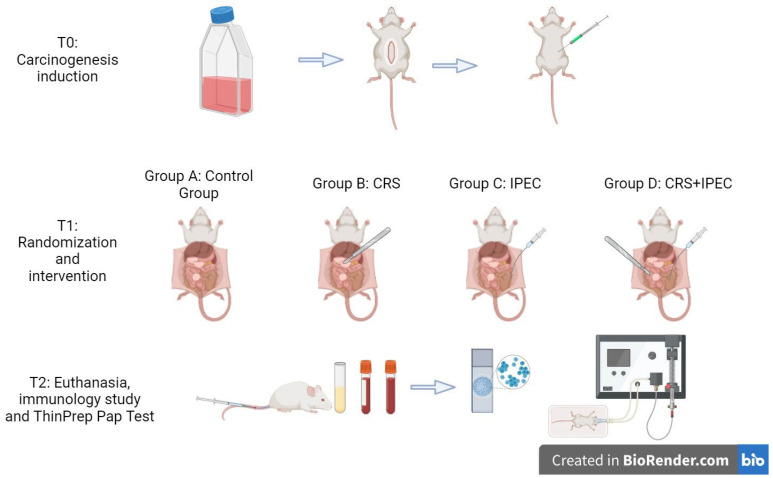
An overview of the experimental design. This figure was created with BioRender.com.

After observation for 28 days, at T2 (Day 56), blood sample collection was performed for an immunology study (IL-6, TNF-α) after anesthesia was administered. Animals were sacrificed by means of CO_2_ inhalation for further investigations. Then, midline laparotomy was performed and ascites was harvested for the cytological study. In case of premature death of a rat, the time and cause of death were registered, and then laparotomy and measurements followed. An overview of the experimental setup is presented in Figure 1.

#### 2.3.1. Flow Cytometry Analysis (FACS) of Serum Cytokines

Levels of TNF-α and IL-6 in serum were determined using a panel kit (AimPlex Biosciences, Pomona, CA, USA) tested via Flow Cytometer Analysis using a BD FACS Calibur system (BD Biosciences, San Jose, CA, USA), according to the manufacturer’s recommendations and instructions. The intra-assay and inter-assay variability in serum cytokine measurements with FACs are the Coefficients of Variability (CVs): <10% and <20%, respectively. Specifically, 45 μL of serum sample and 45 μL beads of the panel kit were mixed and then incubated for 1 h at room temperature. After incubation, 0.5 mL of wash buffer was added, and the samples were centrifuged for 5 min. Samples were incubated first with biotin-conjugated monoclonal antibody (30 min) and then subsequently incubated with streptavidin-conjugated monoclonal antibody (20 min). Finally, wash reading buffer was added to all samples. The data were evaluated using FlowJo software (ver. 7.6; TreeStar Inc., San Carlos, CA, USA).

#### 2.3.2. Peritoneal Cytology

After euthanasia and laparotomy, the peritoneal cytology sample was collected with a suction device from the peritoneal space of rats. Care was taken to avoid any major manipulation before the collection of a single cytology specimen in order to minimize contamination by red blood cells. All ascetic specimens were prepared using the ThinPrep liquid-based cytology preparation system (Cytyc Co., Boxborough, MA, USA). The specimen was centrifuged at 600 g for 10 min, and then the supernatant was poured off carefully. The cell pellet was resuspended and washed with 4 mL of CytoLyt solution. The sample was added to a PreservCyt (Cytyc Co.) solution vial and allowed to stand for 15 min. The vial was then placed in a Cytyc ThinPrep 2000 processor and cells were transferred from the specimen to a glass slide. The slide was fixed in 95% ethanol and stained using Papanicolaou and the diastase–periodic acid–Schiff staining method.

An experienced pathologist was responsible for evaluating the slides. A slide was classified as positive if three-dimensional clusters of malignant cells were present or one malignant cell had a high nuclear cell ratio and over 10-fold enlargement when compared to an adjacent lymphocyte. Atypical findings were classified as negative.

### 2.4. Primary and Secondary Outcomes

The primary outcome is the level of serum IL-6 and TNF-α among groups. Secondary outcomes include peritoneal cytology as positive versus negative.

### 2.5. Sample Size Calculation and Statistical Analysis

The literature includes no previous data regarding the intraperitoneal administration of regorafenib. This is a pilot study conducted to evaluate the effect of IPEC with regorafenib, with or without CRS, on the management of metastatic CRC in a rat model. No sample size calculation was performed and rats were assigned randomly, in a proportion of 1:1:1:1, to 4 groups, each containing 6 animals. The final sample size included 24 specimens.

The measured variables were checked for the normality of their distribution using the Shapiro–Wilk test. Normally distributed, continuous variables were expressed by the arithmetic mean ± standard deviation (mean ± SD), while continuous variables with non-parametric distribution were expressed by the median and intra-quadratic range (median, IQR). Qualitative variables, categorical or ordinal, are presented as numbers and percentages per 100. The confidence interval was set at 95% which means that the differences between the groups were considered statistically significant when *p* < 0.05. To compare the variables in two independent study groups, a t-test was used for parametric distributable data, whereas the Mann–Whitney U test was used for non-parametric distributable data. The Kruskal–Wallis test with Dwass–Steel–Critchlow–Fligner for pairwise comparisons was used for the comparison of the three groups. The comparisons of the independent nominal variables were performed using the chi-squared test, as the expected counts were greater than 5. The statistical analysis of the results was performed using the statistical program Jamovi Version 1.6.18.0.

## 3. Results

In our experiment, 24 rats were included and randomized into the four arms. At T0, carcinomatosis was induced as described. At T1, after metastasis inspection, animals were operated on according to their assigned arm. Two deaths were recorded before T2, and these rats were evaluated prematurely. More specifically, one rat from group B and one from group D died on the fourth postoperative day and fifth postoperative day, respectively, due to peritonitis following an anastomotic leak.

### 3.1. Primary Outcomes

#### 3.1.1. Serum IL-6

**Figure 2:**
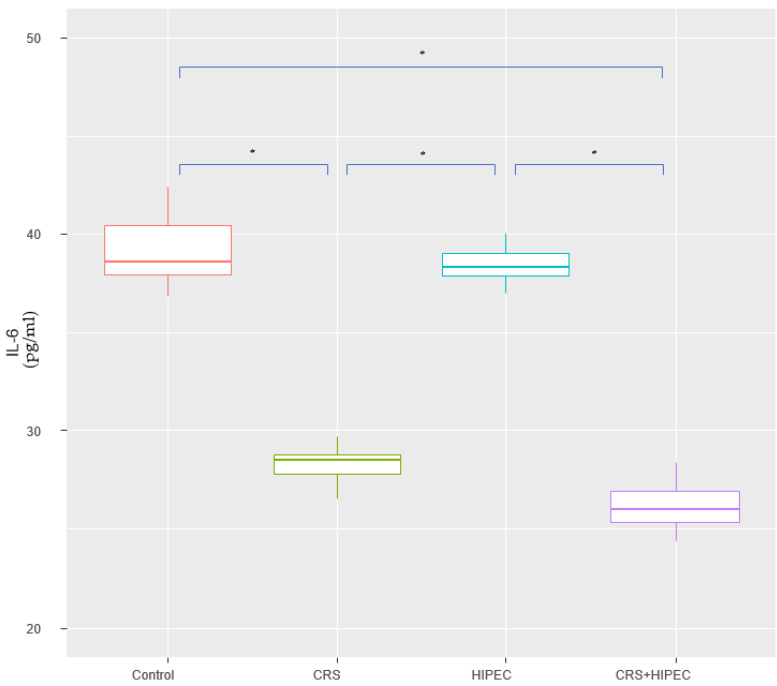
Serum IL-6 levels at T2 (Day 56) (asterisks indicate significant differences). Carcinogenesis induction was performed at T0 (Day 0). After 28 days (T1-Day 28), rats were allocated randomly into 4 groups (group A: control group; group B: CRS only; group C: IPEC only; group D: CRS + IPEC). Regorafenib (10 mg/kg) was used as an IPEC agent. Fifty-six days after carcinogenesis, blood samples were collected for IL-6 level measurement. The confidence interval was set at 95%, which means that the differences between the groups were considered statistically significant when *p* < 0.05. * *p* < 0.05.

At T2, IL-6 levels of groups A, B, C, and D were 39.8 ± 1.69 pg/mL, 28.2 ± 0.981 pg/mL, 38.0 ± 1.06 pg/mL, and 26.9 ± 1.17 pg/mL, respectively. No statistically significant difference was found between groups A vs. C (*p* = 0.092; CI: −0.200; 3.60) and groups B vs. D (*p* = 0.093; CI: −0.200; 2.70). Compared to groups A and C, group D had a lower mean IL-6 level and the difference was statistically significant (*p* = 0.002). Looking into group B versus groups A and C, we found a statistically significant difference regarding the lower IL-6 levels in group B (*p* = 0.002). The results are presented in Figure 2. 

#### 3.1.2. Serum TNF-α

**Figure 3:**
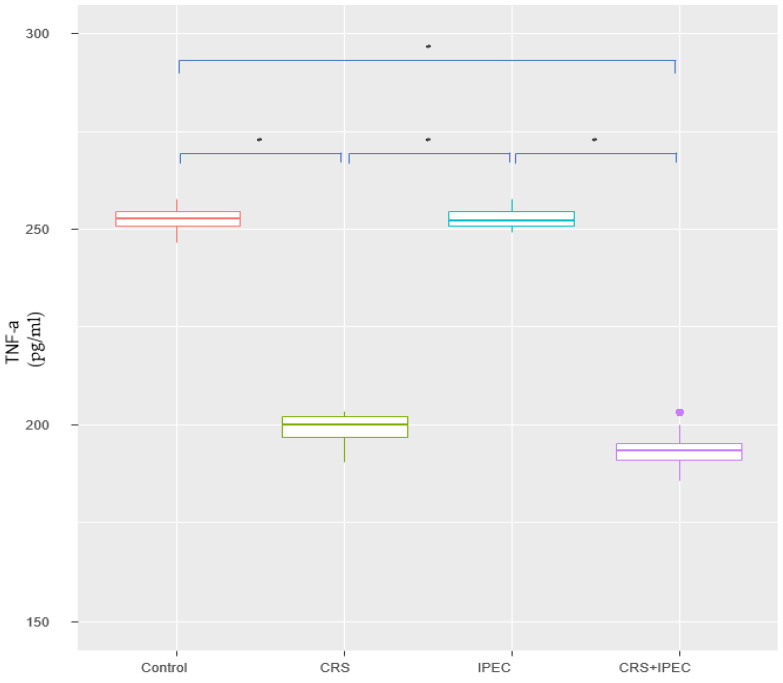
Serum TNF-a levels at T2 (Day 56) (asterisks indicate significant differences). Carcinogenesis induction was performed at T0 (Day 0). After 28 days (T1-Day 28), rats were allocated randomly into 4 groups (group A: control group; group B: CRS only; group C: IPEC only; group D: CRS + IPEC). Regorafenib (10 mg/kg) was used as an IPEC agent. Fifty-six days after carcinogenesis, blood samples were collected for TNF-a level measurement. The confidence interval was set at 95%, which means that the differences between the groups were considered statistically significant when *p* < 0.05. * *p* < 0.05.

At T2, the TNF-α levels of groups A, B, C, and D were 253.0 ± 3.25 pg/mL, 198.0 ± 3.65 pg/mL, 252.0 ± 2.52 pg/mL, and 195.0 ± 4.48 pg/mL, respectively. No statistically significant difference was found between groups A vs. C (*p* = 0.485 > 0.05; CI: −2.00; 5.30) and groups B vs. D (*p* = 0.065 > 0.05; CI: −0.900; 9.70). In addition, serum TNF-α was lower in group D, compared to groups A and C, and the difference was statistically significant (*p* = 0.003). Looking into group B versus groups A and C, we found a statistically significant difference towards lower TNF-α levels in group B (*p* = 0.003). The results are presented in Figure 3.

### 3.2. Secondary Outcomes

#### Peritoneal Cytology

**Figure 4:**
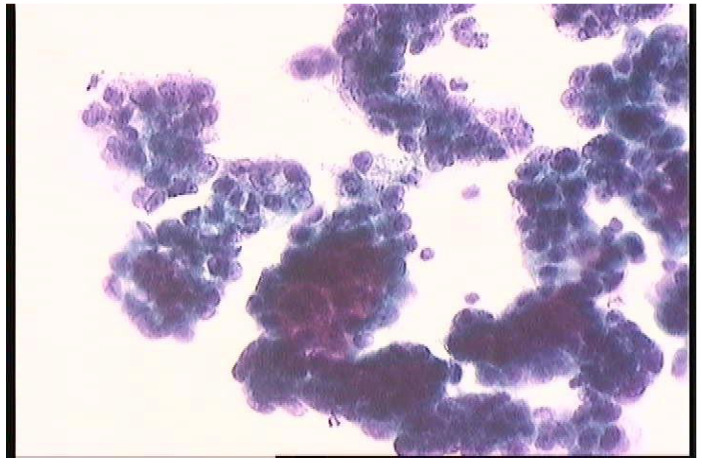
Peritoneal cytology. The malignant cells are arranged in clusters with overlapping nuclei.

**Figure 5:**
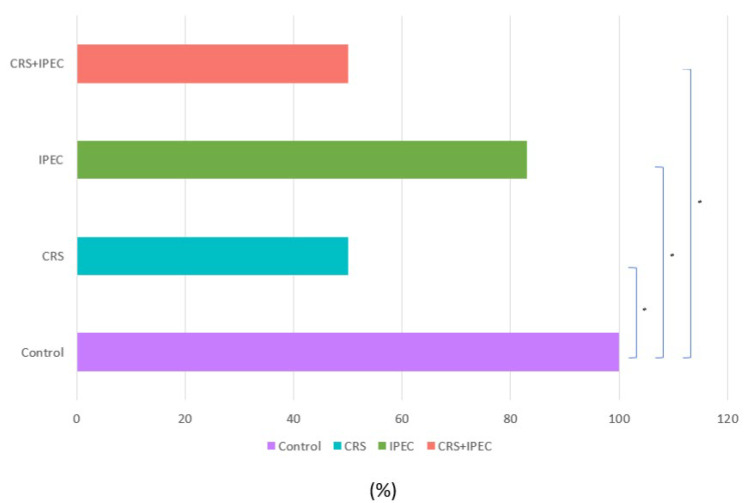
Peritoneal cytology results at T2 (Day 56) (asterisks indicate significant differences). Carcinogenesis induction was performed at T0 (Day 0). After 28 days (T1-Day 28), rats were allocated randomly into 4 groups (group A: control group; group B: CRS only; group C: IPEC only; group D: CRS + IPEC). Regorafenib (10 mg/kg) was used as an IPEC agent. Fifty-six days after carcinogenesis, euthanasia and midline laparotomy were performed. A peritoneal cytology sample was collected with a suction device from the peritoneal space. All ascetic specimens were prepared using the ThinPrep liquid-based cytology preparation system (Cytyc Co., Boxborough, MA, USA). The confidence interval was set at 95%, which means that the differences between the groups were considered statistically significant when *p* < 0.05. * *p* < 0.05.

At T2, positive peritoneal cytology was detected in 6/6 rats (100.0%) in group A, 5/6 rats (83.3%) in group C, and 3/6 rats (50.0%) in groups B and D (Figure 4). Both CRS and CRS + IPEC groups presented a statistically significant difference regarding the response to intervention and the rate of negative peritoneal cytology specimens when compared to the control group (*p* = 0.046 for both comparisons). On the contrary, the combination of CRS + IPEC did not manage to achieve a statistically significant difference to the IPEC group regarding the rate of negative peritoneal cytology specimens (*p* = 0.221), while it led to the same results with the CRS group (*p* = 1). The results are presented in Figure 5.

## 4. Discussion

CRC is the third most common type of cancer and the second most common cause of cancer-related death globally [20]. In 2023 alone, approximately 153.020 new cases will be diagnosed with CRC and 52.550 deaths will be noted from the disease, including 19.550 cases and 3.750 deaths affecting patients under 50 years old [21]. The peritoneum is the third most frequent site of recurrence of CRC after the liver and lungs [20]. Synchronous peritoneal spread is present in 5 to 10% of patients, while up to 20 to 50% of patients with recurrence will present with metachronous PM [22]. New chemotherapeutic agents for systemic therapy, apart from their therapeutic role, are associated with numerous side effects due to toxicity, such as myelosuppression and immunosuppression [23]. The adoption of CRS and HIPEC led to a significant increase in median OS from 6 to 12.5 months [24]. Furthermore, the selective and sustained exposure of the peritoneal surface to high drug concentrations significantly increases the therapeutic effect and reduces systemic toxicity [23]. 

Regorafenib is an orally administered kinase inhibitor, used for the management of patients with refractory metastatic CRC, who previously received fluoropyrimidine-, oxaliplatin-, and irinotecan-based regimens [25]. This constitutes a multi-targeted therapy, affecting multiple aspects of tumor biology [26]. The targeting of angiogenesis is a basic strategy in the development of new agents for metastatic CRC [27]. Regorafenib inhibits angiogenesis via the inhibition of Vascular Endothelial Growth Factor Receptors (VEGFRs), as well as via the inhibition of fibroblast growth factor receptors (FGFRs) 1 and 2, the angiopoietin-1 receptor TIE2, and platelet-derived growth factor receptors (PDGFRs) α and β [25]. The inhibition of VEGFR2 and VEGFR3 also contributes to the remission of tumor metastasis through antiangiogenic and antiproliferative mechanisms, while the inhibition of RAF-1, RET, and KIT pathways leads to reduced cell proliferation and increased apoptosis [25]. Recently, the immunomodulatory role of regorafenib has emerged as it inhibits the CSF1 tyrosine kinase receptor, and, in this way, limits macrophage proliferation [28].

The studies CORRECT and CONCUR were the first phase III clinical trials reporting significantly increased OS with regorafenib monotherapy when compared with placebo for the treatment of treatment-refractory metastatic CRC [29,30]. Later, the CONSIGN study, a phase IIIb study, aimed to investigate the safety profile of regorafenib [31]. The results reported regorafenib-related adverse effects in 91% of participants, with fatigue, HFSR, hypertension, and diarrhea being the most commonly reported effects [31]. To address the systemic toxicity, the REARRANGE trial tried to evaluate the safety and efficacy of two regorafenib dose-escalation approaches in patients with refractory metastatic CRC. The results showed that an intermittent dosing arm presented a significant improvement in the most commonly reported regorafenib-induced adverse effects [32]. Dose escalation was also proposed by the ReDOS study as an effective strategy alleviating the adverse effects of systemic regorafenib administration [33]. 

There is scarce literature regarding the pharmacological activity and pharmacokinetics of regorafenib in experimental models [34]. Zopf et al. suggested that a dose of 10 mg/kg in a mouse model is the dose that leads to similar exposure as that in CRC patients receiving 160 mg/day [35]. These results were later confirmed by a second study which demonstrated that the oral administration of 10–30 mg/kg in mice was both efficacious and well tolerated [36]. Further studies have revealed the low absorption and poor bioavailability of per os administered regorafenib, leading to experimental efforts to ameliorate these parameters [34,37]. Regorafenib’s molecular weight (482 g/mol) and mechanism of action are consistent with the basic principles of IPEC. However, there are no data regarding the ideal intraperitoneal dosage of regorafenib and its consequent bioavailability expressed as the area under the concentration–time curve (AUC) ratio of the peritoneal exposure over the plasma exposure after IPEC [38].

Many experimental models related to peritoneal carcinomatosis from CRC have investigated the role of CRS and IPEC with various regimens, such as MMC, oxaliplatin, irinotecan, gemcitabine, panitumumab, and others. The results were conflicting; however, MMC, irinotecan, and their combination with other agents appeared to be quite effective, providing a survival benefit [39,40,41,42]. Unfortunately, to date, no studies have investigated regorafenib’s intraperitoneal administration and it is only used as a component of oral chemotherapy. The aim of our study was to evaluate its efficacy in a rat model of colorectal-derived PM. In the absence of previous data, we set the dose of intraperitoneal regorafenib at 10 mg/kg, which is the usual dose of oral administration in experimental models.

Previously published data from our study demonstrated the feasibility of the method. Specifically, it was depicted that the intraperitoneal administration of regorafenib at a 10 mg/kg dose was safe for the rats, without any important side effects such as weight loss or lethargy [19]. Moreover, the efficacy of the method was tested via the calculation of the experimental Peritoneal Cancer Index (ePCI) as described by Klaver et al. [41]. Our results suggested that intraperitoneal regorafenib holds an additive effect when combined with CRS [19]. The ePCI of groups A (control), B (CRS), C (IPEC) and D (CRS + IPEC) were 14.3 ± 1.5, 7 ± 2.2, 12.3 ± 2.3, and 5.6 ± 1.9, respectively. The ePCI score was significantly lower in group D compared to all other groups (*p* < 0.01), while the ePCI score of group B was significantly reduced compared to group A and C (*p* < 0.01). In addition, the weight of ascites was measured at T2 and was found to be significantly lower in groups B and D in contrast to groups A and C (*p* < 0.02) [19]. Our present paper focused on immunology changes, especially in the serum levels of IL-6 and TNF-α, in the above experimental rat model with peritoneal carcinomatosis from CRC.

A series of cytokines, including TNF-α and IL-6, have been associated with the differentiation and proliferation of tumor cells and tumor neoangiogenesis in CRC [13]. More specifically, high Il-6 serum levels have been associated with poor OS, since IL-6 enhances tumorigenesis through paracrine and autocrine mechanisms and impairs the role of the anti-tumor immune response [43]. In addition, high serum levels of pleiotropic cytokine IL-6 have been related to an advanced stage of disease and the presence of metastatic foci [44]. Moreover, TNF-α contributes to CRC cell proliferation and plays a significant role in CRC-related cachexia [12,45]. 

In the area of colorectal peritoneal carcinomatosis, it has been stated that IL-6 and TNF-α participate in the peritoneal metastatic cascade by creating a beneficial microenvironment for tumor–mesothelial interactions, thus facilitating the attachment of malignant seeds to distant peritoneal surfaces [14]. Many studies have demonstrated that IL-6 and TNF-a enhance tumor cell adhesion and that this is associated with an increased expression of ICAM-1 and VCAM-1 by mesothelial cells [46,47]. Furthermore, cytokines play an important role at the final stage of peritoneal metastasis, which includes cell proliferation and angiogenesis and is already known to be mediated to a certain extent by IL-6 and TNF-a [14].

In our study, rats undergoing CRS were found to have statistically significantly lower plasma IL-6 levels compared to the control and IPEC groups. Also, levels of IL-6 were significantly lower in the CRS + IPEC group in comparison to the control or IPEC group. Regarding the combined role of IPEC with regorafenib and CRS vs. CRS alone, group D showed a tendency towards lower levels of IL-6, revealing an additive effect of regorafenib combined with CRS, but without statistical significance. Regarding TNF-α, both CRS and CRS + IPEC groups had significantly lower serum levels of TNF-α compared to the control and IPEC groups. However, IPEC with the use of regorafenib combined with CRS tended to present lower levels of TNF-α than CRS alone, but a statistically significant difference was not proven. Lower serum levels of IL-6 and TNF-α in the CRS + IPEC group are in accordance with the significantly lower levels of ePCI score and ascites weight, previously published by our team, enhancing the role of cytokine levels in estimating, or even predicting, peritoneal spread from CRC [19]. To the best of our knowledge, no other experimental study in the literature related to CRC peritoneal carcinomatosis and IPEC has investigated the relevance of the peritoneal tumor burden to the level of cytokines.

Early diagnosis of the presence of extracolonic spread and peritoneal carcinomatosis is of paramount importance, since the administration of neoadjuvant chemotherapy followed by CRS, instead of colonic resection alone, may improve long-term survival rates [48]. In patients with gastric or ovarian cancer, determination of the presence of free peritoneal cancer cells by paracentesis followed by peritoneal cytology constitutes a basic part of the initial patient evaluation [49,50]. With regard to this, the literature contains numerous reports suggesting the role of peritoneal cytology in CRC [51,52]. In addition, Sugarbaker et al. suggested a statistically significant correlation between PCI score and cytology positivity [52]. However, they concluded that peritoneal cytology may not have a role in detecting microscopic peritoneal disease during primary CRC resection, but that it may rather be an indicator of a more advanced extent of peritoneal spread [52]. In addition, Kanellos et al. proposed an estimated accuracy of 85% of positive peritoneal cytology for predicting local recurrence [53]. Based on our previous results, where the CRS + IPEC group presented significantly lower ePCI compared to other groups, a lower cytology positivity rate would be expected [19]. However, the group treated with CRS + IPEC presented the same cytology positivity rates as with CRS, and lower but not significantly lower rates compared to IPEC, probably due to the small sample size. 

Of note, this pilot study also has a series of limitations. The aforementioned results are primitive and based on a small sample size. In addition, the lack of previous literature data, such as the feasibility of intraperitoneal use and appropriate dosage or bioavailability of regorafenib, may result in an inconsistency and should be investigated in the future. Further preclinical studies focused on immunology outcomes as indicators of tumor progression are required, as well as studies investigating the importance of ascites cytology positivity.

## 5. Conclusions

Regorafenib-based IPEC, combined with CRS, may constitute a promising tool against peritoneal carcinomatosis by altering the tumor microenvironment. However, further studies are necessary for establishing the safety and efficacy profile.

## Data Availability

Data are available to any qualified researchers upon request to sbitsiani@gmail.com.
